# Assessing the effect of deficit irrigation and biochar application on soil water depletion, root distribution, and water productivity of cucumber in semi-arid West Texas

**DOI:** 10.3389/fpls.2026.1796736

**Published:** 2026-06-08

**Authors:** Arjun Kafle, Sukhbir Singh, Manpreet Singh, Preetaman Bajwa, Sanjit Deb, Catherine Simpson, Glen Ritchie

**Affiliations:** 1 Department of Plant and Soil Science, Texas Tech University, Lubbock, TX, United States; 2 Kearney Agricultural Research and Extension Center, University of California Agriculture and Natural Resources, Parlier, CA, United States; 3 School of Integrative Plant Science, Cornell University, Ithaca, NY, United States; 4 Department of Agronomy, Iowa State University, Ames, IA, United States

**Keywords:** crop evapotranspiration, irrigation strategy, root plasticity, soil amendment, soil water extraction

## Abstract

Understanding crop root growth and distribution and soil water depletion under water deficit conditions is critical for developing sustainable production practices for cucumber (*Cucumis sativus* L.) in semi-arid regions like West Texas. Therefore, this study’s main objective was to investigate the effect of deficit irrigation (DI) and biochar application on soil water depletion, root growth and distribution, and water productivity (WP) of cucumber. A two-year field study was conducted at Quaker Research Farm, Texas Tech University, Lubbock, TX. A split-plot design was used to randomize four irrigation levels I1 [100% crop evapotranspiration (ETc) throughout the growing season], I2 [80% ETc during early growth (crop establishment to mid-season), 60% ETc during late growth (mid-season to maturity)], I3 (60% ETc during early growth, 80% ETc during late growth), I4 (40% ETc throughout the growing season) and three biochar rates (0, 15, and 20 t/ha) in main-plots and sub-plots, respectively. Results showed a decrease in root length density (RLD) and root surface area density (RSAD) under DI treatments compared to control (I1). RLD was reduced by 24, 4, and 29%, whereas RSAD decreased by 24, 31, and 44% in I2, I3, and I4, respectively, compared to I1. There was greater soil water depletion under the severe DI treatment (I4) without enhanced root plasticity, with some occasional water storage in mild-DI treatments (I2 and I3). The crop water use decreased significantly by 17% in I2, with the least yield penalty of 14% compared to I1. The I2 was the most water productive treatment compared to other DI treatments. Although biochar showed some positive effects on RSAD, it had marginal effects on soil water depletion and water productivity. This study suggests DI strategy optimized soil water depletion by regulating root adaptations or compensatory responses to DI-induced mild to moderate water stress during the growing season, while improving WP for successful cucumber production. It is recommended to test biochar over a longer period (> 2 years) or at higher application rates to better understand its influence on cucumber production and WP in West Texas region.

## Highlights

- Root surface area density (RSAD) reduced more than root length density (RLD) under deficit irrigation treatments compared to control.
- Greater soil water depletion was observed under the severe deficit irrigation regime (I4) having just 40% ETc water replacement before and after mid-season without enhanced root plasticity.
- Deficit irrigation I2 (80% ETc water replacement before mid-season followed by 60% ETc water replacement after mid-season) showed least yield penalty of 14% with crop water use 17% less than full irrigation I1 (100% ETc water replacement).
- Biochar induced effects were marginal on soil water depletion and water productivity and recommend for long term study.

## 1 Introduction

West Texas, a semi-arid region in the Texas High Plains (THP), faces significant challenges related to water scarcity due to frequent drought events accompanied by low and erratic rainfall, as well as high soil evaporation or evapotranspiration rates. This condition increases producers’ reliance on underground water resources such as the Ogallala Aquifer. The higher rate of water withdrawal than the replenishment of the Ogallala Aquifer has caused a depletion of more than 50% from 1935 to 2012 (Haacker et al., 2016). More than 90% of the water withdrawal from the aquifer is used for irrigation (Colaizzi et al., 2009). Continued water withdrawal for irrigated agriculture is expected to increase water footprints, potentially leading to a serious risk of land conversion to dryland farming soon. Hence, to extend the life of the aquifer and for agricultural sustainability, it is imperative to adopt water-conserving irrigation management strategies in the region (Chaves and Davies, 2010).

Deficit irrigation (DI) is one of the most efficient ways to conserve soil vadose zone water in arid and semi-arid regions (Al-Ghobari and Dewidar, 2018). Under DI, irrigation is restricted by supplying less water to plants, but to a degree that can sustain yield and optimize water use efficiency (WUE). The WUE is the measure of the productivity of plants in producing yield based on the amount of water utilized (Bwambale et al., 2022; Wang et al., 2021; Zhang et al., 2022; Zhao et al., 2020). DI has been extensively used in vegetable production; however, results have varied across species and in response to different DI levels (Costa et al., 2007; Singh et al., 2021). Singh et al. (2019) reported that among 134 studies, 44% of the studies showed no significant yield loss in moderate DI compared to full irrigation, while 52% of them exhibited significant yield loss. This variability in results occurred due to varying water extraction patterns and root adjustment of different crops under water-limiting conditions (Debaeke and Aboudrare, 2004; Wasson et al., 2012).

Root growth and distribution have a large role in utilizing the available soil moisture for plant growth. The plant water extraction pattern is greatly determined by the root penetration capacity and root characteristics (Kondo et al., 2000). Soil water depletion and root distribution interact, influencing each other. This means that root water uptake depends on the distribution of the roots, where the absorption of water by the roots alters the available soil moisture content (Parkash et al., 2021b). Root length, root biomass, root thickness, or diameter are some major traits that change under limited soil moisture conditions (Comas et al., 2013). While crops with a deeper rooting system can extract available water from deeper soil depths to ameliorate water stress in plants, crops with shallow root systems are primarily impacted under diminishing water conditions in the soil. When water is limited in the soil profile, it increases the soil mechanical resistance and impedance for root growth and elongation (Hodgkinson et al., 2017). Roots often exhibit enhanced growth in their pursuit of water, especially under moderate soil moisture conditions at field capacity. However, inadequate water availability under progressively soil drying conditions can severely hinder root development and ultimately impact the plant’s growth. Notably, researchers have reported that root adaptations, specifically by regulating root growth and distribution, in response to water stress differ among crop types or crop species (Guan et al., 2015; Sharma et al., 2017) and DI levels (Gheysari et al., 2017; Xu et al., 2016), leading to different patterns of water extraction from the soil and reflecting diverse crop adaptations to water stress conditions. The plant root system is most critical for adaptation against water stress, enabling it to uptake moisture and nutrients. These adaptations can involve changes in root length density, especially across different root diameter classes, root distribution patterns, and the development of a more extensive root system to access water from deeper soil layers. Under DI strategies, it is more critical to determine the ability of crops to efficiently utilize the root zone water content, as this directly relates to water use efficiency or water productivity (WP) for efficient crop production and breeding strategies (Sharma et al., 2014). Furthermore, there has been increasing interest in combining DI with other conservation strategies, particularly the application of soil amendments that minimize the crop yield trade-off, improve WP, and sustain crop production in water-limited agricultural systems.

Biochar, a carbonaceous, porous, and recalcitrant compound, popularly known as “black gold,” has been gaining more attention as a water and soil conserving strategy, mostly in arid and semi-arid agroecosystems (Keller, 2019). For example, Bruun et al. (2014) reported that biochar improved the root growth of barley due to increased water retention in the sandy subsoil. Another study on sweet corn by Singh et al. (2023) demonstrated that root length density increased under hardwood biochar treatments in sandy clay loam soil. The findings of these studies indicate that biochar can be beneficial in improving root growth in different soil textures. Biochar has been used in different crops, notably in the region where water scarcity is a prominent problem. Specially, there is a growing interest in using biochar under DI in vegetable crop production (Agbna et al., 2017; Alkhasha et al., 2019; Ebrahimi et al., 2021). Abewoy (2018) further highlighted the importance of investing in soil amendments, such as biochar, for growing vegetable crops, as they can provide a high value return per cost for small growers. Besides the positive effects of biochar, biochar has also shown some negative impacts in vegetable crops due to potential risk of contaminants that comes with biochar amendments (Xu et al., 2025). These contrasting results of biochar effects in vegetable crops highlight the need for more research- not only to understand aboveground performance but also to explore belowground response of crops. While most studies are limited only to examine aboveground effects (Ebrahimi et al., 2021; Murtaza et al., 2024; Obadi et al., 2023), reports on the effects of DI and biochar on root distribution, water depletion, and WP of vegetable crops are still limited in number.

Cucumber, a moderately tolerant to water-stress vegetable crop, has great potential to be used as an income-generating fresh vegetable due to its increasing demand in West Texas. Cucumbers require an ample amount of water to produce fresh fruits, which makes growing it in a semi-arid region like West Texas challenging. Due to its deep root system, cucumber can access water from deeper soil layers, enabling the crop to maintain its water status and survive when the water availability in the upper soil layers is limited, thereby adapting under limited water conditions. A two-year field study under seasonal DI with 80% crop evapotranspiration (ETc), 60% ETc, and 40% ETc showed that cucumber water use efficiency can remain comparable between full irrigation and all DI treatments despite reduced root growth under water limitations (Parkash et al., 2021b). Their findings suggest that cucumbers can maintain efficient water utilization even under DI-induced water stress conditions, likely due to compensatory mechanisms such as adjusting root water uptake and growth, as well as physiological processes. The findings further underscore the potential relationship between root modification and WP, and various DI management strategies can be implemented to optimize this relationship in regions like West Texas facing water limitations in irrigated vegetable crop production. Additionally, a metanalysis of 43 different studies performed by Gao et al. (2020) concluded that there was a statistically significant positive response of biochar application on WUE of various crops. Although these studies identified the implications of DI and biochar on increasing WUE independently, the applicability of integrating both conservation practices in vegetable crops like cucumber is yet to be clearly understood. Further, majority of these studies only focus on aboveground crop adaptability disregarding the below ground phenomenon which plays a crucial role in overall crop resiliency. Many studies skip time and labor-intensive root extraction disregarding the initiation point of response towards abiotic stress like water stress especially in the deep-rooted crops like cucumber. Research is needed to expand our knowledge of how cucumbers adapt water stress under a combined practice of DI and biochar amendment to maintain efficient water utilization and enhanced yield by regulating root growth and distribution, and potentially by modifying the root system. With increasing number of local vegetable producers, information on root adjustment and water uptake for greater water productivity under reduced irrigation and soil amendment practice will help them understand the belowground mechanism of crop endurance under drought stress. Hence, the objective of this study is to assess the effect of DI and biochar applications on soil water depletion, root distribution, and WP of cucumber grown under semi-arid conditions of West Texas.

## 2 Materials and methods

### 2.1 Experimental site description

A two-year (2021 and 2022) field study was conducted at Quaker Research Farm, Texas Tech University, Lubbock, Texas (33° 36′ 18″ N, -101° 54′ 26″ W and 992 m above mean sea level). The climatic condition of the region is semi-arid, with an average annual rainfall of 469 mm and average annual evapotranspiration of 1500 mm (TAMU, 2023). The average annual maximum, minimum, and mean temperatures of the region were 23.3, 7.8, and 15.6 °C, respectively. The soil type of the study site is sandy clay loam (0–10 cm soil depth), characterized by a distribution of sand, silt, and clay at 55%, 11%, and 33%, respectively, and saturated hydraulic conductivity of 50–60 cm/day (Kafle et al., 2025a, b).

### 2.2 Cultivation practices and experimental design

The field was plowed with a tractor-mounted disk plow to incorporate plant residues up to 15 cm soil depth during the early spring of both years. The raised beds, 50 cm wide × 15 cm high, were prepared with sub-surface drip irrigation lines situated at 30–40 cm soil depth. Fertilizer URAN 32 (NPK 32-0-0) (Nutrien Ag Solution, Loveland, CO) was fertigated once before planting at a rate of 80 kg/ha based on soil test analysis. “Bristol” cultivar of cucumber (Rupp Seed Inc., Wauseon, OH) was planted on June 10 and June 6 in 2021 and 2022, respectively, using a tractor-mounted four-row planter at a rate of 45,000 seeds/ha. The cultivar is second generation hybrid with higher yield potential and greater stress tolerance level, mostly adaptive to warm, dry, and water limited regions like West Texas. Oak tree-derived hardwood biochar (Wakefield™ BioChar, Valdosta, GA) was spread once before the first-year planting on March 18, 2021, and incorporated to a soil depth of 0–20 cm using a tractor-mounted rotary tiller. The physicochemical properties of biochar have been described in Singh et al. (2022).

A weather station (Davis Wireless Vantage Pro2, Hayward, CA) was installed near the experimental field to record weather parameters. The reference evapotranspiration (ETo) was calculated using the Penman-Monteith method (Allen et al., 1998), and crop evapotranspiration (ETc) was further calculated by multiplying ETo by the growth stage-based crop coefficient (Kc) (Zotarelli et al., 2010). The growth stage-based Kc values were K_c initial_ = 0.45 [0–20 Days After Planting (DAP)], K_c crop dev._ = 0.70 (21–50 DAP), K_c mid_ = 0.90 (51–90 DAP), K_c late_ = 0.75 (91–105 DAP) (Kafle et al., 2025b). Mostly the DI is targeted to meet certain degree of crop specific evapotranspiration (ETc) which represents the water demand of the crop. The transition of differential irrigation from early growth to late growth stage was made at mid-season (55 days after planting) without any gradual transition to impose the sudden change in water availability for crops to adapt. Irrigation treatments were applied weekly to restore the ETc from the previous week from crop establishment until one week before harvest.

The experiment was laid out in a split-plot design, with irrigation levels as the main factor and biochar rates as the sub-plot factor, with four replications. There were four irrigation treatments I1[100% crop evapotranspiration (ETc) throughout the growing season], I2 [80% ETc during early growth (crop establishment to mid-season), 60% ETc during late growth (mid-season to maturity)], I3 (60% ETc during early growth, 80% ETc during late growth), I4 (40% ETc throughout the growing season) and three biochar treatments at rate of 0 t/ha (no biochar), 15 t/ha, and 20 t/ha. As there were not any previous studies to recommend a specific range of biochar rates in the region, our previous studies with different types of biochar (softwood and hardwood) at 13 t/ha prompted us to utilize these rates considering the cost of biochar for economical use. Each irrigation zone had eight raised beds, each controlled by a different irrigation regulator. The drip tape of 2.5 cm diameter had emitters spaced out at 60 cm discharging at 1.21 liters of water per hour. Under each irrigation zone, biochar treatments were allocated to each plot, which was 6 m long and 4 m wide. Each plot was separated by a 1.5 m alley within the irrigation zone. In 2021, irrigation water amounts of 341, 249, 306, and 185 mm were applied to I1, I2, I3, and I4, respectively. Due to hot and dry weather conditions in 2022, amounts applied were 452, 353, 374, and 283 mm, respectively. The cucumbers were harvested based on marketable size standards every week from a pre-determined 6 m^2^ area of each plot, and the total yield was obtained by adding fruit weights from all the harvests. In 2021, there were seven harvests, whereas in 2022, there were six harvests.

### 2.3 Soil water content and water productivity

The volumetric water content (VWC, cm^3^/cm^3^) was measured using a capacitance probe (PR2/6 Profile Probe, Delta-T Devices Ltd., Cambridge, UK) at soil depths of 10, 20, 30, 40, 60, and 100 cm. The measurements were made on a weekly basis after 32 DAP and 35 DAP in 2021 and 2022, respectively, once the crop establishment had been achieved and differential irrigation was started. The access tubes were installed between two plants in the center of each plot. In both years, the equivalent depth of water (mm) at each soil depth was determined by multiplying the measured VWC by the soil depth (Bhattarai et al., 2020; Dhakal et al., 2019). The soil water depletion (mm) was obtained by subtracting the equivalent depth of measured at the end of the given period from the equivalent depth of water at the beginning of that measurement period. Soil water depletion accounts for whether soil moisture has been depleted or has been stored at different depths. Therefore, soil water depletion was observed for 32–46 DAP, 46–60 DAP, 60–80 DAP, 80–96 DAP in 2021 and 35–50 DAP, 50–70 DAP, 70–90 DAP, 90–103 DAP in 2022. The seasonal change in soil water storage (ΔS) was calculated by subtracting the VWC from all the depths at harvest from VWC at the initial measurement (32–96 DAP for 2021 and 35–103 DAP in 2022). The ΔS was converted to depth units based on the soil depth of VWC monitoring.

The seasonal crop water use (ET, mm), which accounts for the actual water loss from a specific crop field in its current condition considering loss, gain and storage component and was estimated for both growing seasons using the following water balance equation:

(1) ET=P+I+CR±ΔS−D−R

where P= precipitation, I = irrigation, CR= capillary rise, ΔS = change in seasonal water storage in 100-cm soil profile (32–96 DAP for 2021 and 35–103 DAP in 2022), D = drainage below the root zone, and R = surface runoff. Because the ground water table was very deep (>50 m) and had no contribution to root zone water influx, CR was considered null. Also irrigation was a sub-surface drip delivering water in a controlled rate and the field was flat with slope of 0-1%, the D and R were considered negligible due to the soil characteristics and for simplification of seasonal water balance estimation for DI strategies, as well as physical and resource constraints associated with field measurements (Bajwa et al., 2025; Singh et al., 2023).

Water Productivity (WP) was obtained by the following formula:

(2) WP=Yield(kg/ha)/ET(mm)

where ET was given by Equation 1. WP for each irrigation was computed using Equation 2.

### 2.4 Root core sampling, root extraction, and root image analysis

A stainless split-core sampler, measuring 60 cm in length (two 30 cm splitting cups attached with a coupler) and 5 cm in diameter (AMS, American Falls, ID, USA), was used to collect the root samples in the soil core at the end of the growing season in both years (Bajwa et al., 2025). Previous studies have indicated that 60 cm root sampling depth relates to cucumber rooting potential in semi-arid soils (Parkash et al., 2021b; Şimşek et al., 2005). The split core was pushed to a depth of 60 cm in the soil using a heavy-duty slide hammer. The samples were taken 2.5-3.0-cm away from the base of the plant, perpendicular to the plant row. After that, the core sampler was extracted from the ground using a hi-lift jack. Undisturbed soil in the split core was cut at 10-cm intervals along the length to represent the samples for 0-10, 10-20, 20-30, 30-40, 40-50, and 50-60-cm soil depth. Samples were kept in zip lock bags and stored at 4 °C in the refrigerator until the roots were washed. Root washing was performed by placing the soil core sample with roots in a fine mesh strainer, with another strainer positioned below to collect any roots that might escape from the upper strainer. A gentle stream of pressurized water was administered to the upper strainer, washing the soil out of the roots from both strainers with the soil core samples. The roots were collected from both strainers using forceps to store in a 50-ml Falcon tube containing approximately 40 ml of a 15% (v/v) ethyl alcohol solution. The roots were then scanned using a flatbed EPSON scanner (EPSON V800, Reagent Instruments, Quebec, Canada) to obtain the image, and the root analysis was performed using WinRHIZO Pro Version 2020a software (Reagent Instruments, Quebec, Canada). The root length density (RLD, cm/cm^3^), root surface area density (RSAD, cm^2^/cm^3^), and root fineness classification (% of total root length belonging to 0-0.5, 0.5-1, 1-1.5, >1.5 mm root diameter classes) were obtained (Parkash et al., 2021b). While RLD provides information about the extent of the root system’s exploration of the soil for understanding water and nutrient uptake, RSAD provides information about the potential for water and nutrient uptake at the root-soil surface where exchange processes occur.

### 2.5 Statistical analysis

The collected data were analyzed using analysis of variance (ANOVA) with a split-plot design in R (Team, 2013) version 3.5.2 using Agricolae package version 1.2-8 (de Mendiburu, 2015). For water depletion at each interval and root, a split-split-plot design was adopted, taking irrigation, biochar, and depth as the fixed factors. Any interaction (irrigation or biochar with depth) was again analyzed separately using a split-plot design for each soil depth. Analysis was done separately for both years. The separation of means was done using the Fisher’s least significant difference (LSD) approach at a 5% level of significance. Graphs were prepared using SigmaPlot software version 14 (Systat Software, San Jose, CA).

## 3 Results and discussion

### 3.1 Weather conditions and rainfall

During June, the average temperature was relatively cooler in 2021 compared to 2022 (**Table 1**). July and August of 2021 were also relatively cooler than 2022, as the maximum temperature was 3-3.5 °C lower in 2021 compared to 2022. During the last month of September, the maximum, average, and minimum temperatures were higher in 2021 compared to 2022. The solar radiation for June and July was lower but higher in August and September in 2021 compared to 2022 (**Table 1**). The RH data showed that 2021 was less dry compared to 2022 due to the higher minimum RH values for most of the time period in 2021 (**Table 1**). There was rainfall of 53.6 mm in June, which alleviated the water stress on crops from the early period of the crop in 2021 (**Table 1**). No rainfall events occurred during the early period of the crop in 2022, which put the crops under transpiration stress from the beginning of the crop cycle (Kafle et al., 2025b). Rainfall was well-distributed in July (54.9 mm) and August (41.4 mm) of 2021, but in 2022, the precipitation was only 2.8 mm in July and was concentrated mainly in August with 106.1 mm. There were not many rainfall events in September of both years, but the amount was comparatively higher in 2022 compared to 2021.

**Table 1:** Monthly maximum (max), minimum (min) and average (avg) temperatures, solar radiation (SR), maximum (max) and minimum (min) relative humidity (RH), and rainfall during the 2021 and 2022 growing seasons in Lubbock, TX.

Months	Temperature (°C)						SR (MJ/m^2^)		RH (%)				Rainfall (mm)	
	Max		Min		Avg				Max		Min			
	2021	2022	2021	2022	2021	2022	2021	2022	2021	2022	2021	2022	2021	2022
June	41.9	41.0	15.3	14.0	27.2	28.0	23.3	27.2	99.8	93.4	4.3	12.0	53.6	0.3
July	35.2	39.4	17.2	20.0	26.1	29.3	23.2	25.2	97.8	78.0	27.0	11.0	54.9	2.8
August	36.0	39.5	14.1	13.3	26.0	26.1	21.8	19.2	98.4	99.4	17.1	16.0	41.4	106.1
September	36.0	33.7	13.7	10.0	26.2	23.5	20.4	20.0	95.3	98.0	33.5	16.9	0.1	5.1

### 3.2 Root growth and depth distribution of roots

RLD within 0–60 cm soil depth did not differ significantly among DI levels in 2021 but varied significantly in 2022 (**Table 2**). This year-to-year differences in RLD indicated that climatic variability can have influence on soil root profile because the previous year had more rainfall than later year impacting crop belowground adjustment. When compared to I1, RLD was reduced by 23% and 21% in I2 and I4, respectively, but increased by 11% in I3 in 2021 (**Table 3**). In contrast, RLD decreased in all DI levels compared to I1 in 2022. The decrease in RLD was 25%, 19%, and 37% in I2, I3, and I4, respectively, compared to I1 in 2022 (**Table 3**). The difference in RLD in 2022 can be due to more dryness and fewer rainfall events during the cucumber growing season (**Table 1**). Similarly, the previous reports have emphasized the effect of weather conditions on root growth and development (Calleja-Cabrera et al., 2020; De Moraes et al., 2018; Sharma et al., 2017; Zhang et al., 2009). The RLD in I3 was least affected compared to other DI treatments in both years, showing that 60% ETc followed by 80% ETc water replacement can contribute to enhancing root length in cucumbers. Early-season water stress on plants, which likely impacts root development and early growth, prompts them to explore water in the soil profile, leading to a prioritization of more root growth. Once the extensive root system has been established, mild water stress at later stages (after mid-season) will not hinder the root system as the one of the possible reason is that plant undergoes morphological adaptations linked to memory of pre-exposure to water stress (Souza et al., 2016). Interestingly, I2 had a comparable RLD with I3, which suggests that, particularly in cucumber, earlier mild water stress (i.e., 80% ETc followed by 60% ETc) can induce cucumber root adjustment that facilitate crop adaptations and drought acclimation. These roots adjustments and crop adaptability under some degree of water stress could possibly linked to the signaling effect of abscisic acid (ABA) as this hormone can stimulate root growth and coordinate with other hormones for root architectural modification (Ranjan et al., 2022).

**Table 2:** Test of significance (p-values) for root length density (RLD), root surface area density (RSAD), root diameter classes based on % of root length (0–0.5 mm, 0.5–1mm, 1–1.5 mm, >1.5 mm) under deficit irrigation and biochar rates during the 2021 and 2022 growing seasons in Lubbock, TX.

	RLD		RSAD		Root classes based on the % of root length							
					0-0.5 mm		0.5–1 mm		1-1.5 mm		>1.5 mm	
	2021	2022	2021	2022	2021	2022	2021	2022	2021	2022	2021	2022
Irrigation (I)	0.13	*	***	**	0.35	0.35	0.42	0.14	0.46	0.25	0.70	0.79
Biochar (B)	0.56	0.58	**	*	0.47	0.11	0.33	*	0.49	0.30	0.41	0.99
Depth (D)	***	***	***	***	***	***	***	***	***	***	***	***
I×B	0.07	0.19	**	0.81	0.80	0.74	0.62	0.59	0.36	0.98	0.69	0.62
I×D	***	***	***	***	0.38	0.15	0.10	*	0.81	0.27	0.87	0.48
B×D	0.57	0.80	***	*	0.48	0.66	0.39	0.90	0.49	0.34	0.37	0.43
I×B×D	*	0.31	***	**	0.30	0.35	0.28	0.44	0.59	0.21	0.69	0.90

*, **, *** denote significant difference/interaction at p ≤ 0.05, p ≤0.01, and p ≤0.001, respectively.

**Table 3:** Effect of deficit irrigation and biochar rates on root length density (RLD), root surface area density (RSAD), and root diameter classes in the 60 cm soil profile during the 2021 and 2022 growing seasons in Lubbock, TX.

Treatments	RLD (cm/cm^3^)	RSAD (cm^2^/cm^3^)	Root classification (diameter classes based on % of the total root length)			
			0–0.5 mm	0.5–1.0 mm	1–1.5 mm	>1.5 mm
2021						
Irrigation (I)						
I1	0.57a	0.09a	76.31a	12.57a	7.78a	3.32a
I2	0.44a	0.06b	75.96a	12.96a	8.15a	2.91a
I3	0.63a	0.06b	82.31a	9.66a	5.77a	2.24a
I4	0.45a	0.05b	78.45a	12.19a	6.87a	2.46a
Biochar (B)						
0 t/ha	0.55a	0.06b	79.77a	10.95a	6.41a	2.85a
15 t/ha	0.50a	0.07a	76.66a	12.69a	7.47a	3.16a
20 t/ha	0.51a	0.07a	78.35a	11.90a	7.54a	2.19a
Depth (D), cm						
10	1.03a	0.10a	89.62a	6.83c	2.7e	0.84d
20	0.61b	0.78b	82.41b	10.54b	5.28d	1.76cd
30	0.51c	0.067c	80.08bc	11.54b	6.17cd	2.19bc
40	0.38d	0.056d	77.01cd	12.31b	7.73bc	2.93bc
50	0.33de	0.046e	73.17d	15.02a	8.62b	3.17b
60	0.27e	0.038f	67.27e	14.85a	12.35a	5.51a
2022						
Irrigation (I)						
I1	0.52a	0.07a	69.13a	16.85a	9.15a	4.85a
I2	0.39bc	0.06b	72.87a	14.02ab	7.91a	5.18a
I3	0.42b	0.05bc	71.07a	15.48ab	7.49a	5.94a
I4	0.33c	0.04c	75.77a	12.54b	7.31a	4.36a
Biochar (B)						
0 t/ha	0.40a	0.04b	75.30a	12.50b	7.03a	5.14a
15 t/ha	0.43a	0.06a	71.57a	15.35a	8.01a	5.05a
20 t/ha	0.42a	0.06a	69.75a	16.32a	8.86a	5.05a
Depth (D), cm						
10	0.69a	0.08a	81.91a	10.98e	3.41c	3.68cd
20	0.58b	0.07b	80.73a	11.77de	5.02c	2.46d
30	0.38c	0.05c	73.00b	13.91cd	8.27b	4.80bc
40	0.33cd	0.05c	71.70b	14.67bc	7.84b	5.77b
50	0.26de	0.05c	65.42c	17.35ab	11.19a	6.01ab
60	0.24e	0.04d	60.48c	19.66a	12.08a	7.77a

Mean values followed by different alphabets within a column and a specific factor denote significant differences at p ≤ 0.05.

Biochar had no effect on RLD in both years of the experiment (**Table 2**). RLD differed significantly among various soil depths (p<0.001) (**Table 2**), where RLD gradually decreased with increasing soil depth (**Table 3**). This is due to increasing soil impedance and resistance for root growth at deeper soil depths under water stress (Cairns et al., 2004). Out of the total sampled depth (0–60 cm), 66-69% of RLD was found in the 0–30 cm range, while 31-34% was present in the 30–60 cm soil depth. Across two years, we observed that RLD at 0–30 cm ranged 71% for I1, 67% for I2, 68% for I3 and 63% for I4 where RLD at 30–60 cm was accounted only 29% for I1, 33% for I2, 32% for I3, and 37% for I4, respectively. Our study is in line with Parkash et al. (2021b), where 68-75% of the total RLD was present in the upper soil profile (0–30 cm) while remaining 25-32% of the total RLD was in deeper soil profile (30–60 cm) in cucumber. Our finding is also in agreement with previous studies, which found that tomato and melon root lengths were more concentrated at shallower soil depths than at deeper depths (Miller et al., 2013; Zotarelli et al., 2009).

**Figure 1:**
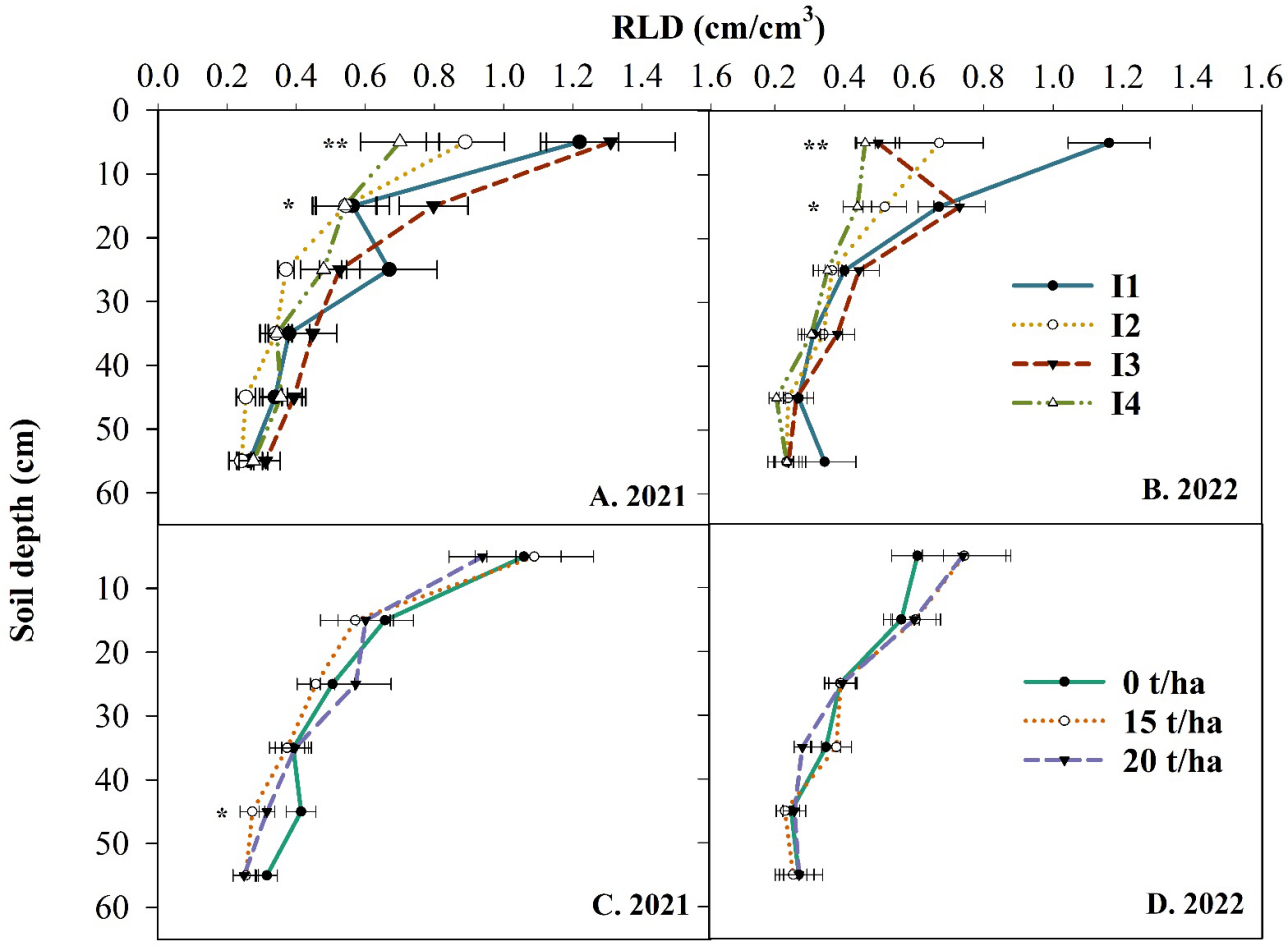
Effect of deficit irrigation **(A, B)** and biochar rates **(C, D)** on root length density (RLD) in 60 cm soil profile during the 2021 and 2022 growing seasons in Lubbock, TX. * and ** denote significant difference at p ≤ 0.05 and p ≤0.01 respectively. The horizontal lines in each depth for each treatment denote the error bars.

There was no significant interaction between irrigation and biochar; however, a significant interaction was observed between irrigation and soil depth for RLD in both years (**Table 2**). The RLD varied among irrigation levels only at shallower depths (0–20 cm) in both years (**Figures 1A, B**). It was observed that RLD under I2 and I3 remained higher than I4 at a soil depth of 0–20 cm. However, RLD under I3 showed unmatching response at 0–10 cm soil depth in both years which was the major driving factor for overall RLD change. This implies that under limited precipitation condition, irrigation at or below 60% ETc during its early to mid-growth phase can impair surficial root growth and this damage may not be recovered even if irrigation was increased to 80% ETc water replacement after mid-season. Beyond 20 cm soil depth, RLD did not vary significantly among irrigation levels, suggesting that water had limited impact on root length at deeper depths. Our results align with the study by Parkash et al. (2021b), which found that RLD did not vary throughout the soil profile in cucumber, except for a significant difference observed at 0–20 cm in our study compared to 0–40 cm in their research. This could be due to the difference in seasonal vs. growth stage-based DI strategy, which alters water availability in the soil profile affecting root exploration. There was no significant interaction between biochar and soil depth for RLD in either year (**Table 2**) (**Figure 1C**). Biochar did not influence RLD in any depth in either year, although 0 t/ha had significantly higher RLD at a 40–50 cm soil depth in 2021 (**Figures 1C, D**) due to some anecdotal measurements. However, significant interaction occurred between irrigation, biochar, and depth only in 2021 (**Table 2**), which are not presented in the manuscript as interaction was inconsistent and did not alter the main conclusion, and especially three-way interaction is less important for the first growing season following a single, topsoil incorporation strategy for biochar application.

The main and interaction effects on RSAD determined within 60 cm soil depth were significant in both years (**Table 2**). With decreasing water levels, RSAD decreased by 33% in I2, 33% in I3, and 44% in I4 in 2021, and by 14%, 29%, and 43% in 2022, respectively, compared to I1 (**Table 3**). These results demonstrate that RSAD is more sensitive to irrigation than RLD. RLD and RSAD are influenced by root diameter distribution. A root system with many fine roots (high fine root RLD) may have a greater RSAD. Li et al. (2022) reported that RSAD varied when RLD did not change at a 9–12 cm horizontal distance from the base of rice seedlings due to an allelopathic effect for weed suppression at a 0–5 cm soil depth. This could be because plants alter the surface area more rapidly than root length to explore water and nutrients in the soil, thereby enhancing resource acquisition efficiency. The roots extending up to 40 cm soil depth were mainly affected. Biochar also significantly affected RSAD (**Table 2**), with a 17% and 17% increase in 2021 and a 50% and 50% increase in 2022, respectively, in both 15 and 20 t/ha biochar applications compared to the control (**Table 3**). RSAD followed the same pattern as RLD, gradually decreasing with increasing soil depth. The significant interaction between irrigation and biochar in both years (**Table 2**) showed the highest RSAD at 15 t/ha under I1; however, it was the least at 15 t/ha under I4 in 2021 and 0 t/ha under I4 in 2022, respectively (data not shown). This suggests that biochar application can enhance root surface area, facilitating crop adaptation with increased water availability (Singh et al., 2023). Also, the partial effects of biochar on RSAD could be explained by biochar induced positive changes in root architecture by promoting root branching and improving soil properties for greater resource uptake to which surface area plays greater role than root length (Abiven et al., 2015). A previous study in cucumber seedling establishment in a biochar amendment trial in soilless media also suggested that, cucumber is prone to alter its RSAD than RLD to utilize space, water and nutrients to better acclimatization and adaptability (Kafle et al., 2023).

**Figure 2:**
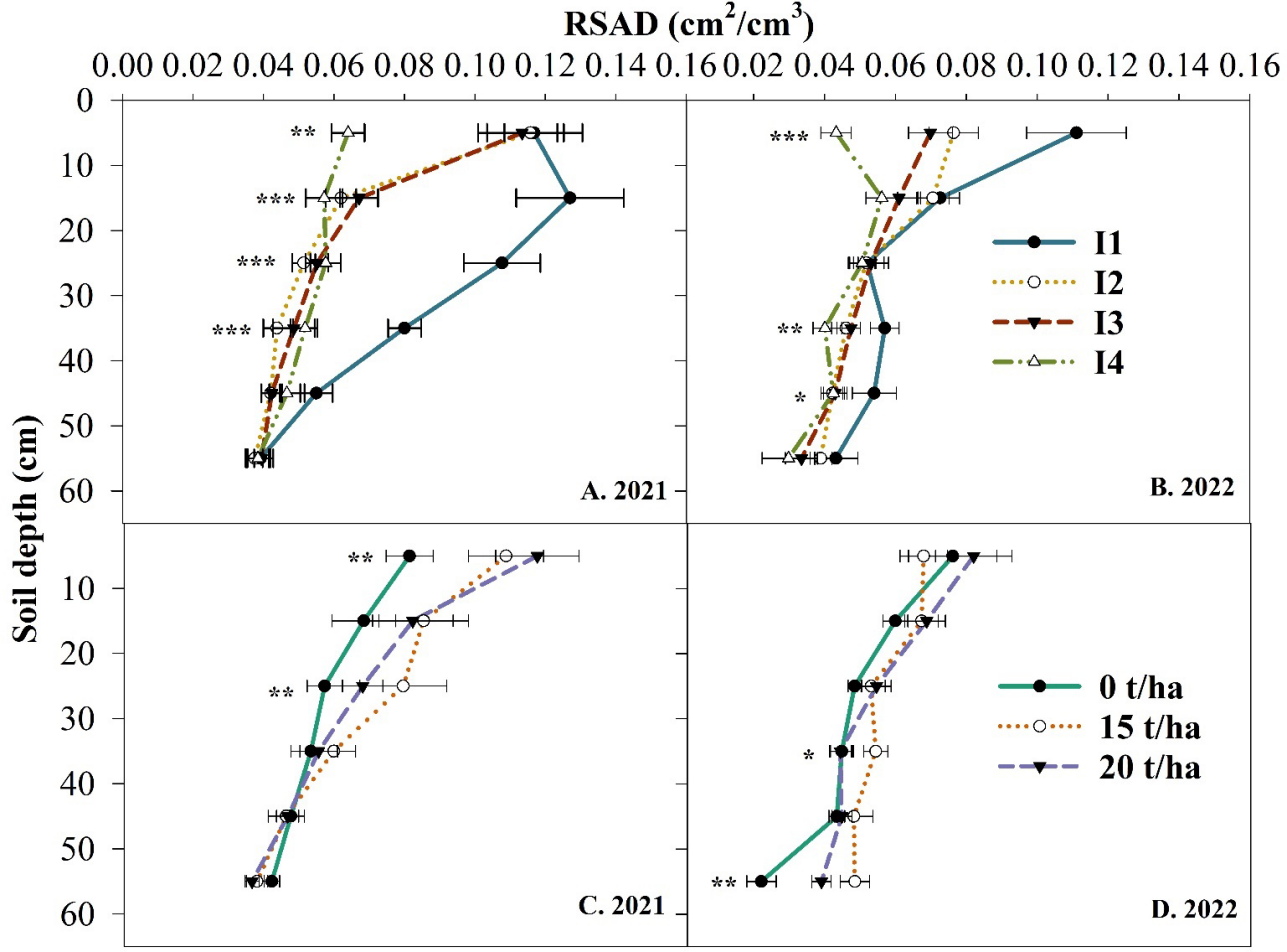
Effect of deficit irrigation **(A, B)** and biochar rates **(C. D)** on root surface area density (RSAD) in 60 cm soil profile during the 2021 and 2022 growing seasons in Lubbock, TX. *, **, *** denote significant difference at p ≤ 0.05, p ≤0.01, and p ≤0.001, respectively. The horizontal lines in each depth for each treatment denote the error bars.

The significant interaction between irrigation and soil depth (p<0.05) (**Table 2**) revealed that I1 had a comparatively greater RSAD than the other DI treatments at all soil depths in both years (**Figure 2**). In 2021, the significant difference in RSAD occurred at a soil depth of 0–40 cm whereas it occurred at only 0-10, 30-40, and 40–50 cm in 2022 (**Figures 2A, B**). Our results agree with the findings of Parkash et al. (2021b), who reported that the RSAD was highest at DI level of 100% ETc and lowest at 40% ETc, where the significant differences occurred up to a 0–40 cm soil depth. In our study, RSAD values under I2 and I3 for most soil depths fell somewhere between the RSAD observed under I1 and I4. Our study also followed a similar pattern having significant difference at an additional 10 cm soil depth (40–50 cm). The numerical values of RSAD observed in the upper soil depths were also found to be consistent in the deeper 50–60 cm depths; however, the difference in RSAD was non-significant at 50–60 cm. This suggests that, throughout the soil profile (0–60 cm), the sensitivity of RSAD retards its growth due to water limitations for plants that are directly associated with lower soil water content conditions (Parkash et al., 2021b; Singh et al., 2023). Across two-year study results, the critical layers can be 0–10 cm (upper) and 30–50 cm (lower) where cucumber plant can likely to adapt their root system to explore soil layers for water when subjected to water stress imposed by DI strategy. A significant interaction between biochar and soil depth (p < 0.05) (**Table 2**) was observed, with lower RSAD in 0 t/ha biochar treatment for most of the soil profile in both years (**Figures 2C, D**). In 2021, RSAD was significantly lower in 0 t/ha biochar treatment at both 0–10 and 20–30 cm soil depths compared to other biochar-amended treatments (**Figure 2C**). But in 2022, 0 t/ha and 20 t/ha biochar treatments had significantly lower RSAD at soil depth 30–40 cm, but at deeper soil depth of 50–60 cm, RSAD significantly decreased in 0 t/ha, with the highest in 15 t/ha biochar treatments (**Figure 2D**). These differences with inconsistent results can come from soil heterogeneity or sampling artifacts. While predicting the exact extent of biochar’s influence down to 60 cm depth is complex, it is plausible that biochar incorporated at 15 cm could affect RSAD at that depth. The interconnected nature of the root system allows for the transport of various substances and signals via its vascular tissues. Therefore, the conditions or influences at upper depth roots or root distribution can affect the root system and its functionality at deeper soil depths (Zegada-Lizarazu and Monti, 2019). The three-way significant interaction between irrigation, biochar and soil depth (p<0.05) did not show a consistent pattern in RSAD in both years where it was highest under I2 at 20 t/ha biochar treatment at the topsoil depth of 0–10 cm in 2021, but it was highest in I1 at 0 t/ha and 20 t/ha biochar treatment at 0–10 cm sol depth (data not shown).

Root classification (0-0.5, 1-1.5, >1.5 mm) was not significantly affected by the main effects of irrigation and biochar or their interaction, except in relation to soil depth (**Table 2**). The percentage of the total root length under the 0-0.5 mm diameter class was high in the upper soil depth and low at deeper soil depths in both years (**Table 3**). A similar pattern in fine root diameter class 0-0.5 mm was reported only in the first year in melon (Sharma et al., 2014) and in the second year in cucumber (Parkash et al., 2021b). However, in our study, the results regarding the higher percentage of fine diameter class roots (0-0.5 mm) were consistent for both growing seasons, suggesting that cucumber produces roots of narrower diameters (fine) in shallower depth under various DI levels combined with biochar amendment. This is further explained by the gradual increase in percentage of broader diameter classes (1-1.5 and >1.5 mm, tap roots) with increasing soil depth (**Table 3**). The differences in root diameter class 0.5–1 mm did not vary significantly among irrigation levels, but numerically, they were the highest in I1 and the lowest in I4. Among biochar rates in 2022, the root diameter class of 0.5–1 mm was observed to be the highest when biochar was applied at rates of 15 and 20 t/ha, and conversely, the lowest instance of this root diameter class occurred with no biochar application (0 t/ha). For the soil profile, the 0.5–1 mm root diameter class was most prevalent at depths of 40–50 and 50–60 cm, while the lowest prevalence was observed in the upper soil depths (i.e., 0–10 and 10–20 cm) (**Tables 2**, **3**).

### 3.3 Soil water depletion

**Figure 3:**
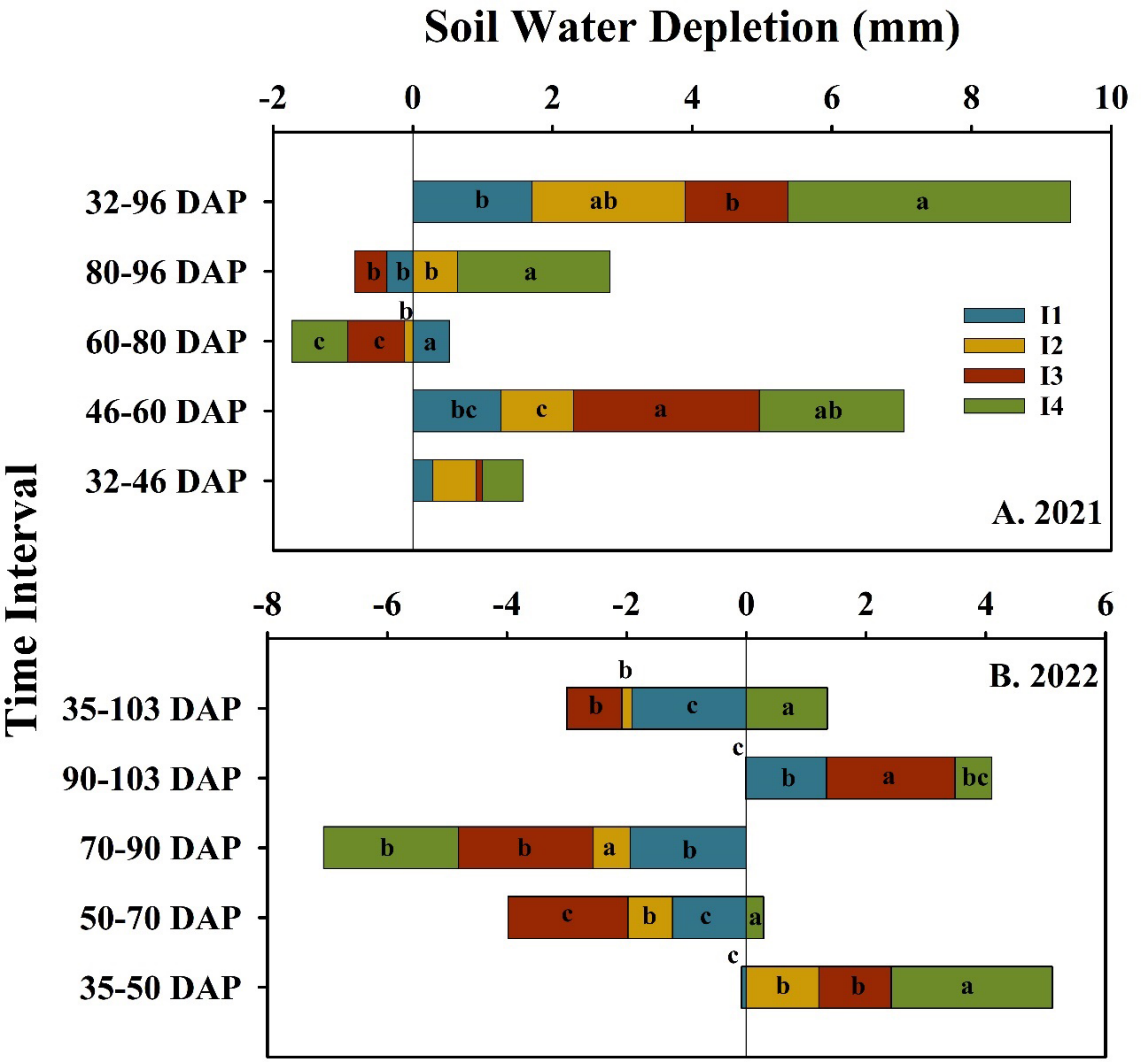
Effect of deficit irrigation on total soil water depletion at 100 cm soil depth occurring at different time periods during the 2021 **(A)** and 2022 **(B)** growing seasons in Lubbock, TX. Different alphabets within horizontal stacked bars denote significant difference at each measurement time interval at p ≤ 0.05.

Soil water depletion varied significantly among irrigation levels at 46-60, 60-80, 80-96, and 32–96 DAP in 2021 and at 35-50, 50-70, 70-90, 90-103, and 35–103 DAP in 2022 (**Figure 3**). In 2021, at 46–60 DAP, I3 and I4 showed significant depletion of water from the 100 cm soil profile. This may indicate that cucumber plants under severe water stress tend to deplete more water from the soil to meet the increasing transpiration demand of the crop as it proceeds towards mid-season (Parkash et al., 2021b). Severe DI if depletes more water to keep up with transpiration then it satisfies the purpose of DI to induce root adaptations or compensatory mechanism but if the transpiration is not adequately met then it will negatively affect soil water availability, depleting more water resulting in larger water deficit or drier soil, especially for extreme DI levels. At 60–80 DAP, the heavy rainfall added excess water to the soil, leading to water storage in other irrigation treatments than I1, leaving more water in the soil profile than the extraction capacity of the developed roots in DI treatments. Later, during 80–96 DAP, plants under I4 depleted the largest amount of water to support growth and survival under water stress at a later stage. Overall, the seasonal soil water depletion during 36–96 DAP was the highest in I4 and the least in I3. Compared to I1, the DI treatments I2 and I4 exhibited 29% and 138% greater water depletion, respectively. In contrast, I3 showed the lowest water depletion (13% less) for the 32–96 DAP period in 2021 (**Figure 3**).

In 2022, I4 depleted more water from the soil profile during 35–50 and 50–70 DAP compared to other treatments. During 70–90 DAP, water was stored in all irrigation treatments due to continuous rainfall events that added water to the soil profile. Deficit irrigation I3 had the highest soil water depletion during the 90–103 DAP period, and I4 showed more water depletion from the soil profile during the 35–103 DAP period. Compared to I1, irrigation I2 and I3 showed less water storage (91% and 52% less, respectively), while I4 depleted 170% more water in 2022 for a 35–103 DAP period (**Figure 3**). Our study is in agreement with previous studies, which indicate that soil water depletion increases as water limitation rises in the soil profile (Bhattarai et al., 2020; Katuwal et al., 2020; Prieto et al., 1999). Soil water depletion gets rapid in the condition where transpiration demand is high due to high temperature accompanied by less soil water available which is evident in I4 irrigation regime. Biochar did not significantly affect soil water depletion in either year but showed that biochar can store more water than unamended treatment in a seasonal period (**Supplementary Table 1**). There was also no interaction between DI and biochar rates for soil water depletion (**Supplementary Table 1**).

**Figure 4:**
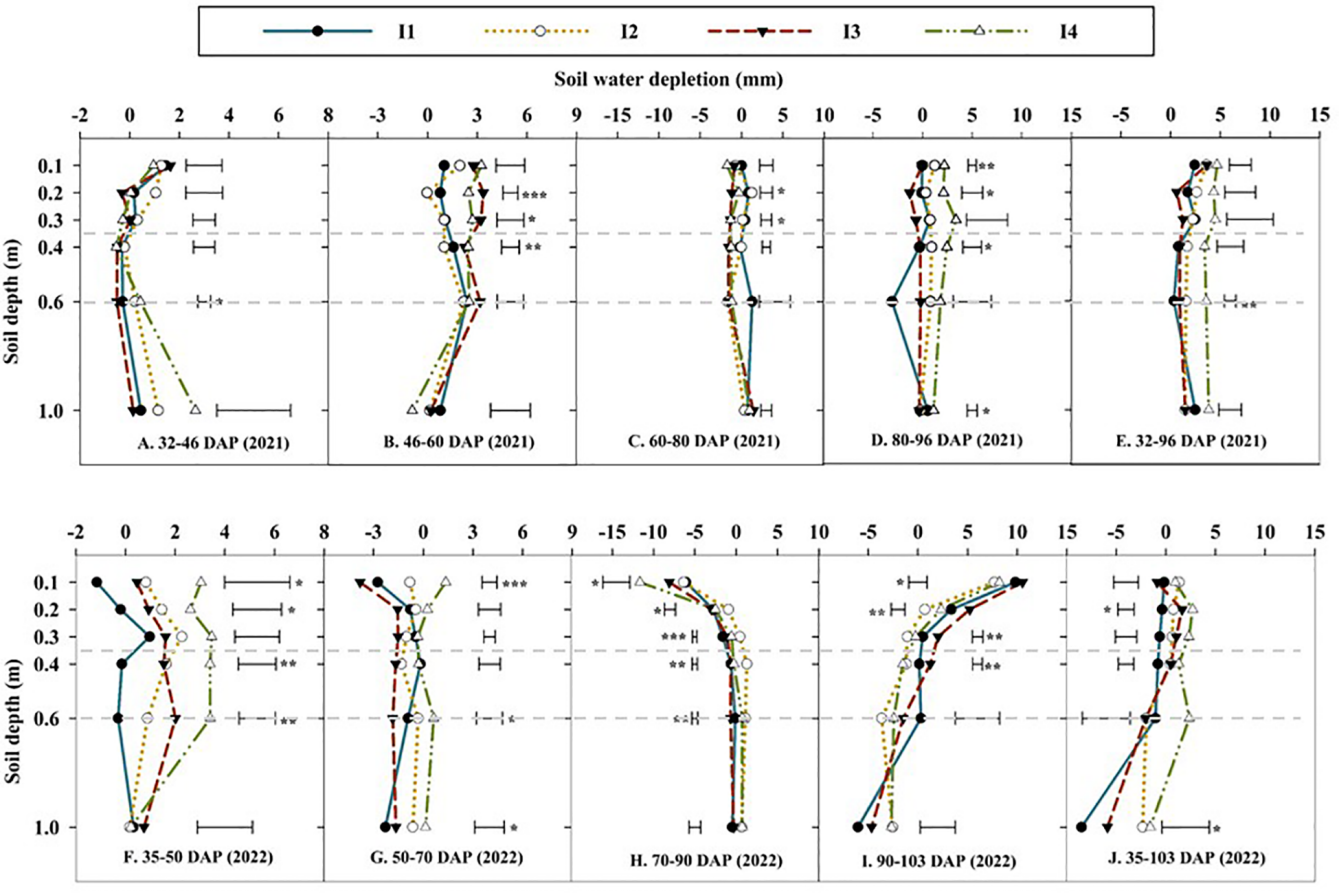
Effect of deficit irrigation on soil water depletion at 10, 20, 30, 40, 60, and 100 cm soil depth during the 2021 **(A-E)** and 2022 **(F-J)** growing seasons in Lubbock, TX. The upper and bottom dotted lines shown on each graph denote the depth of drip tapes and root measurements, respectively. *, **, *** denote significant difference at p ≤ 0.05, p ≤0.01, and p ≤0.001, respectively. The horizontal lines at each depth denote least significant difference (LSD).

A significant interaction between irrigation and soil depth was observed for soil water depletion (**Figure 4**). In 2021, significant differences in soil water depletion occurred among irrigation treatments mostly within the 0–60 cm soil profile (**Figures 4A–C, E**), except at 80–96 DAP (**Figure 4D**). The soil water depletion was the highest in I4 at 60 cm depth for the 32–46 DAP period (**Figure 4A**), while the highest soil water depletion was observed in I3 at 20 and 30 cm depths, and in I4 at 40 cm for 46–60 DAP (**Figure 4B**) in 2021. The soil water depletion was the highest in I1 and I2 at 20 and 30cm for 60–80 DAP (**Figure 4C**), and in I4 at 10, 20, 40 and 100 cm for 80–96 DAP (**Figure 4D**), and in I4 at 60 cm for 32–96 DAP (**Figure 4E**). Due to the stressful weather conditions in 2022, significant differences in soil water depletion occurred throughout the 100 cm soil profile, except in certain depths and time periods, among irrigation treatments. The soil water depletion was the highest in I4 at 10–60 cm for 35–50 DAP although significant difference did not occur at 30 cm (**Figure 4F**), in I4 at 10, 60 and 100 cm for 50–70 DAP (**Figure 4G**), in I2 at 10–60 cm for 70–90 DAP (**Figure 4H**), in I3 at 10–40 cm for 93–103 DAP (**Figure 4I**), and in I4 at 20 and 100 cm for 35–103 DAP (**Figure 4J**) in 2022. From these results, it can be inferred that the severe deficit irrigation (I4) had the lowest water application and the greatest water depletion throughout the soil profile. This can be supported by the amount of water available in the I4 for roots to grow and proliferate, thereby supporting plant survival (**Figures 1**, **2**). Although the RLD and RSAD were the lowest for I4 at topsoil depths (0–10 and 10–20 cm), it increased significantly or even remained comparable with higher irrigation levels at the soil depth below 20 cm, with a few exceptions. The soil water depletion, occurring around 30 cm or immediately below 30 cm, where the plants are most likely to be adapted to water stress and support root growth, can be attributed to the placement of drip lines at that depth. Hence, more water was depleted under severe water stress due to root modifications, resulting in a significant increase in root densities. Despite some root adaptations, I4 DI likely fails to provide adequate soil water conditions to return the required amount of root zone water storage that is readily available to the plant during various stages. So, DI strategy like I4 should be regulated or adopted using a threshold based on allowable depletion in this study. The insignificant differences in different root diameter classes among irrigation levels signify root adaptation through longitudinal and cross-sectional proliferation under DI, especially in the soil depth surrounding the primary source of water for the plant, which can promote the development of deeper root systems. Notably, maintaining similar root growth while depleting the maximum amount of water when sufficient soil moisture is present in the root zone reflects an effective DI strategy, as intended in our study. Our study agrees with the findings of Kang et al. (2000), who reported that root growth and development were enhanced by imposing DI in maize through alternate furrow irrigation. Hence, root morphology and distribution help crops adapt by sensing ecological variations and utilizing the available water from the soil profile (Ali et al., 2019; Wang et al., 2011).

### 3.4 Evapotranspiration, yield, and water productivity

Irrigation treatments had significant effects on the total seasonal crop water use (ET) in both years (**Table 4**). Due to longer drier periods and higher irrigation amounts in 2022, the ET was higher in all irrigation treatments compared to 2021. The ET values in I2, I3, and I4 were 18%, 7%, and 28% lower in 2021 and 16%, 13%, and 27% lower in 2022, respectively, compared to I1. Our finding is in agreement with previous reports where ET was the highest in 100% ETc and the lowest in 40% ETc (Parkash et al., 2021b; Singh et al., 2023). A similar pattern was also observed in a study by Bhattarai et al. (2020) where ET remained low for the lowest (50 mm) and high for the highest (350 mm) irrigation levels for forage sorghum, pearl millet, and corn in the West Texas region. This pattern occurred because of the greater amount of water available for evapotranspiration at higher irrigation levels compared to the lowest irrigation levels. It is also due to the highest ΔS in I4 and the lowest in I1, which allows more water to be used in I1. The higher soil water depletion in I4 in both years still had the lowest ET because of the lowest irrigation amount among irrigation levels in both years (Parkash et al., 2021b). Biochar did not significantly influence ET, which is consistent with Singh et al. (2023), who reported that biochar had a marginal effect on soil water retention while growing sweet corn due to less pronounced effect in soil hydraulic properties.

**Table 4:** Effects of deficit irrigation and biochar rates on the total crop water use (ET), yield, and water productivity (WP) during the 2021 and 2022 growing seasons in Lubbock, TX.

Treatments	ET (mm)		Yield (kg/ha)		WP (kg/ha/mm)	
	2021	2022	2021	2022	2021	2022
Irrigation (I)						
**I1**	501a	555a	48,933a	32,618a	98a	59a
**I2**	412c	467c	41,423b	28,357a	101a	61a
**I3**	465b	483b	38,828b	28,875a	84b	60a
**I4**	359d	405d	23,022c	18,113b	64c	45b
p value	<0.001	<0.001	<0.001	<0.001	<0.001	0.01
Biochar (B)						
**0 t/ha**	435a	480a	36,908a	25,854a	84a	53a
**15 t/ha**	433a	477a	37,978a	27,188a	87a	57a
**20 t/ha**	435a	476a	39,268a	27,930a	89a	58a
p value	0.77	0.37	0.71	0.47	0.72	0.40
**I×B**	0.51	0.22	0.93	0.74	0.92	0.72

Note: Mean values followed by different alphabets within a column denote significant difference at p ≤ 0.05.

Irrigation treatments significantly influenced yield in both years (**Table 4**). Compared to I1, there were 15%, 20%, and 53% yield reductions in I2, I3, and I4, respectively in 2021. These yield reduction values were 13%, 11%, and 44% in 2022. Previous studies have shown that the yield of cucumber decreases with decreasing water availability under DI (Abd El-Mageed et al., 2018; El-Garawany and Albaloushi, 2015; Zinivand et al., 2020). Similar yield penalty was observed under DI grown other cucurbits. Muskmelon 50% yield reduction was obtained when only 50% evapotranspiration was met at six-days interval compared to a full irrigation treatment (Kirnak et al., 2005). Another study with melons showed that 50% ETc water replacement irrigation resulted 30% yield decline compared to 100% ETc irrigation (Sharma et al., 2014). Qiang et al. (2024) suggested that watermelon yield was higher under water sufficient irrigation treatment compared to water stressed irrigation regimes in two cropping patterns. Yin et al. (2025) found that pumpkin yield reduced by 7.9% and 17.4% when irrigation volume was reduced to 450 m^3^/ha and 375 m^3^/ha compared to 525 m^3^/ha. These studies suggest that cucurbits can yield depression when extreme water stress irrigations approach is imposed and hence intermediate water stress irrigation strategy needs to be explored. It should also be noted that I2 and I3 had comparable yields to I1 in 2022. Alomran and Luki (2012) found that when 80% ETc was applied at different growth stages except mid-season, a comparable yield to full irrigation was obtained. Similar results were also obtained by Parkash et al. (2021a), who reported comparable yield at 80% ETc compared to full irrigation. This is because DI involving 80% ETc irrigation level could optimized the water availability for plants to sustain the cucumber plant growth under water stress conditions at critical growth stages, especially in flowering stage (Potop, 2011). Hence, the narrow yield penalty in I2 during 2021 was due to the supplement of 80% ETc water replacement compared to 60% and 40% ETc in I3 and I4 until mid-season when the cucumber produced flowers. The excessive water stress in the early period of cucumber growth in I3 did not recover when irrigation was increased to 80% ETc later after mid-season. Until mid-season, cucumber growth was generally rapid, producing many fertile flowers at the nodes. However, water stress during this period led to flower abortion and subsequent yield reduction (Kafle et al., 2025b). Under irrigation I2, the water available for root uptake was effectively utilized to produce a higher yield compared to other DI treatments during the 2021 crop season (**Table 4**). Notably, similar water extraction from the soil profile could be the most plausible explanation attributed to a similar yield for I2 and I3 treatments in 2022. The roots were also modified based on water availability under I2 and I3, making these irrigation management strategies more adaptive than the extreme DI level (**Figures 1**, **2**). Biochar did not significantly influence the yield of cucumber, although it was numerically higher than the unamended (i.e., 0 t/ha) control. Biochar influenced the RSAD (**Figure 2**), but it might not be sufficient to bring about a significant improvement in yield. Also, the water depletion under the biochar application (i.e., 15 t/ha and 20 t/ha) was comparable to that of the control with no biochar application. However, yield under biochar treatments were numerically higher than unamended treatment, which can also suggest that biochar might have retained more water and could have been made available for plants uptake to increase yield to some extent. Hence, longer period research studies are needed to depict a more pronounced effect on yield when biochar-induced benefits in the soil. Additionally, there was no significant interaction between irrigation and biochar for yield of cucumber.

WP in I3 and I4 was significantly reduced by 14% and 39% in 2021. However, in 2022, only I4 significantly reduced the WP by 24% compared to I1 (**Table 4**). The comparable WP in I2 with I1 was attributed to higher yields and lower water use compared to other DI treatments in both years (**Table 4**). Our results are in agreement with previous findings, which show that mild water stress at 80% ETc increased the WUE in cucumbers compared to full irrigation (Alomran and Luki, 2012; Parkash et al., 2021b). Accordingly, imposing subtle water stress by employing DI levels can increase WP. In our experiment, DI strategy using DI level of 80% ETc followed by DI level of 60% ETc produced higher WP. It signifies that imposing mild water stress until mid-season could enhance the WP of cucumber. In another study, the WUE of tomato was found to be enhanced when 75% ETc irrigation was applied at the vegetative or fruiting stage compared to other treatments (Al-Harbi et al., 2015). It can also be visualized from the soil water depletion and root explorations relationship, where water was extracted from the soil (**Figure 3**) by influencing RLD and RSAD (**Table 3**). In 2022, all the DI had comparable WP with I1. This could occur, because, under DI, crops increase WUE by lowering the unproductive water loss and efficiently utilizing available water (Al-Harbi et al., 2015; Geerts and Raes, 2009; Steduto and Albrizio, 2005). However, other environmental factors can influence the success and efficiency of these strategies. The drier conditions in 2022 affected the fruit yield even though the water applied through irrigation was higher than in 2021, resulting in a reduction in WP in 2022. WP did not vary significantly among biochar treatments (**Table 4**). However, numerically, 15 t/ha and 20 t/ha increased WP by 4% and 6% in 2021 and 8% and 9% in 2022, respectively, compared to the control (0 t/ha). The lack of biochar induced impact on WP can possibly count as one of the limitations of this study due to short period of observation time (2 years), as growers might want to know the long-term impact of the biochar application on water efficiency. Also, the rates that were tested in this study could have been low to see more pronounced effect of biochar and probably require higher rate. There was no significant interaction between irrigation and biochar on the WP observed in cucumber for both years.

Looking at the bigger picture, in regions like semi-arid, irrigation availability is becoming scarce, which puts thousands of local producers at risk as their livelihood is solely dependent upon the water resources and on-farm crop productivity. Especially with vegetable producers, the choice of crops, timing of irrigation, and farm inputs account for very important decision. This research was conducted with grower involvement with day-to-day research activity demonstrating how cucumbers respond to strategic application of irrigation and biochar, discovering the underneath phenomenon that many studies avoid, which holds its strength. Further, the uniqueness of this study also lies in its attempt to diversify cropland in a cotton-dominated region with fresh and profitable vegetable production business. Although assumptions of negligible influence were made for capillary rise, drainage, and surface runoff, however, more robust and precise calculation may give better picture on the water balance under different irrigation regimes. Additionally, the soil water depletion was measured independently at each interval across the season which limits the insight into temporal dynamics under various irrigation regimes. Hence, exploration on the treatment effects over time will give another way of interpreting water dynamics under united irrigated production system in future. Considering the benefits, the study shows strong potential and tendency to serve a transferable practice to similar agroclimatic regions where water is scarce and requires irrigation scheduling technique based on water sensitivity of the crops. Biochar marginal impacts on crop performance observed in this study could be attributed to limitation of study in a single location, short term (two year-duration) trial, and moderate rates. Due to absence of established rate guidelines, the rates tested in this study were derived to reflect economic feasibility in this production system which turned out to be below the threshold required to elicit immediate measurable responses. However, it carries a huge potential as a soil amendment because once it gets mixed and settles well with soil particles, it will contribute to soil stability due to improvement in soil properties. Further, many previous studies suggest that biochar shows positive response in crops with aging effect which further signifies the need for long term evaluation. We also encourage to apply locally available biochar where plant residues are plenty that can lower farm costs and builds soil resiliency with stable compound that lasts long.

## 4 Conclusions

This study utilized DI and biochar to optimize cucumber water productivity in West Texas. Root distribution in cucumber displayed a decreasing RLD and RSAD pattern as the water limitation in the soil, imposed by DI strategies, became more severe. Over two years of study, RLD decreased by 24%, 4%, and 29%; however, RSAD showed a larger decrease of 24%, 31%, and 44% in I2, I3, and I4, respectively, compared to I1. With some exceptions, DI restricted the root growth throughout the 60 cm soil profile. Soil water depletion was more severe when irrigation water was limited to a greater extent (I4) reflecting its incapability of favoring water limited adaptation mechanism or the enhanced root plasticity. The ET decreased significantly by 17%, 10%, and 27% in I2, I3, and I4, respectively compared to I1 across two years. The least yield reduction in I2 significantly improved WP and maintained comparable WP value compared to I1. DI strategy, especially I2, optimized soil water utilization by regulating root adaptations in response to DI-induced mild to moderate water stress during the growing season improving WP. Results indicate that replacing water demand up to 80% ETc until mid-season, followed by 60% ETc replacement during the rest of growing season, can give satisfactory yield while saving water in irrigation suggesting being water-productive irrigation strategy for cucumber production in West Texas. Biochar significantly promoted RSAD but had minimal effect on soil water depletion and WP of cucumber. This study suggests testing biochar under DI for more than two years and with higher rate for greater insights on cucumber adaptability to biochar-induced soil conditions.

## Data Availability

The original contributions presented in the study are included in the article/**Supplementary Material**. Further inquiries can be directed to the corresponding author.

## Supplementary material

Supplementary Table 1 Effect of biochar rates on soil water depletion at different time periods during the 2021 and 2022 growing season in Lubbock, TX. Mean values followed by same alphabets within a column denote non-significant difference among treatments at p ≤ 0.05.
