# Supplementary material for: Assessing the effect of deficit irrigation and biochar application on soil water depletion, root distribution, and water productivity of cucumber in semi-arid West Texas
Source: Front Plant Sci. 2026 Jun 8;17:1796736. doi: 10.3389/fpls.2026.1796736 (PMC13284756; doi:10.3389/fpls.2026.1796736)
Supplement: Supplementary Table 1 — Effect of biochar rates on soil water depletion at different time periods during the 2021 and 2022 growing season in Lubbock, TX. Mean values followed by same alphabets within a column denote non-significant difference among treatments at p ≤ 0.05. [file Table1.docx]

**Supplementary Table 1:**

Effect of biochar rates on soil water depletion at different time periods during the 2021 and 2022 growing seasons in Lubbock, TX.

| **Year** | **Treatments** | **Soil Water Depletion** | | | | |
| --- | --- | --- | --- | --- | --- | --- |
|  |  | **(mm)** | | | | |
| **2021** |  | **32-46 DAP** | **46-60 DAP** | **60-80 DAP** | **80-96 DAP** | **32-96 DAP** |
|  | **Biochar (B)** |  |  |  |  |  |
|  | **0 t/ha** | 0.15a | 2.05a | -0.61a | 0.88a | 2.47a |
|  | **15 t/ha** | 0.46a | 1.62a | -0.50a | 0.10a | 2.70a |
|  | **20 t/ha** | 0.57a | 1.60a | -0.28a | 0.50a | 2.41a |
|  | p value | 0.25 | 0.23 | 0.42 | 0.11 | 0.77 |
|  | **I×B** | 0.53 | 0.98 | 0.95 | 0.32 | 0.51 |
| **2022** |  | **35- 50 DAP** | **50-70 DAP** | **70-90 DAP** | **90-103 DAP** | **35-103 DAP** |
|  | **Biochar (B)** |  |  |  |  |  |
|  | **0 t/ha** | 1.04a | -0.67a | -1.64a | 1.25a | -0.01a |
|  | **15 t/ha** | 1.32a | -1.20a | -1.74a | 1.05a | -0.57a |
|  | **20 t/ha** | 1.40a | -0.90a | -1.93a | 0.77a | -0.66a |
|  | p value | 0.44 | 0.1 | 0.47 | 0.57 | 0.37 |
|  | **I×B** | 0.26 | 0.2 | 0.81 | 0.42 | 0.22 |

Note: Mean values followed by same alphabets within a column denote non-significant difference among treatments at p≤0.05.
